# “Bubbly heart,” a case report of Morgagni hernia delayed diagnosis in patient with Down syndrome: The hernia is in the details

**DOI:** 10.1016/j.radcr.2025.04.108

**Published:** 2025-05-23

**Authors:** Valerio D’Agostino, Giulia Valente, Emanuela Federico, Fiorentino Mondillo

**Affiliations:** a “Umberto I” Hospital of Nocera Inferiore, Radiology Department, Nocera Inferiore, Italy; b Department of Advanced Biomedical Sciences, University of Naples Federico II, Naples, Italy; c "Maria Santissima Addolorata" Hospital of Eboli, Radiology Department, Eboli, Italy

**Keywords:** Emergency radiology, Congenital disease, Conventional radiography, Computed tomography, Pediatric radiology

## Abstract

Morgagni hernia represents a rare form of congenital diaphragmatic hernia (2%-5%), characterized by a defect in the anterior and retrosternal diaphragm. It can be associated with other congenital anomalies, especially in conditions like Down syndrome. Symptoms are often nonspecific, and diagnosis can be missed. Imaging plays a main role in diagnosis. We describe a case of a young boy with Down syndrome suffering from fever and shortness of breath. In the appropriate clinical scenario, it is important to consider this entity among the differential diagnoses of Down syndrome patients with respiratory disease.

## Introduction

Morgagni hernia (MH) is a variant of diaphragmatic hernias characterized by a defect in anterior and retrosternal locations on the right side of the diaphragm: it is the result of an embryological defect in the septum trasversum between the lateral aspect of the diaphragm and the anterior chest wall [1,7]; other types of hernia are Bochdalek one, in which the defect is posterolateral, hiatal hernia with a defect at the esophageal hiatus, and paraesophageal one, where the defect is found near to esophageal hiatus and herniated viscera are displaced in the lower retromediastinum along the esophagus path. It is a rare condition representing the 2% to 5% of all congenital diaphragmatic hernias [2].

## Case report

**Fig. 1:**
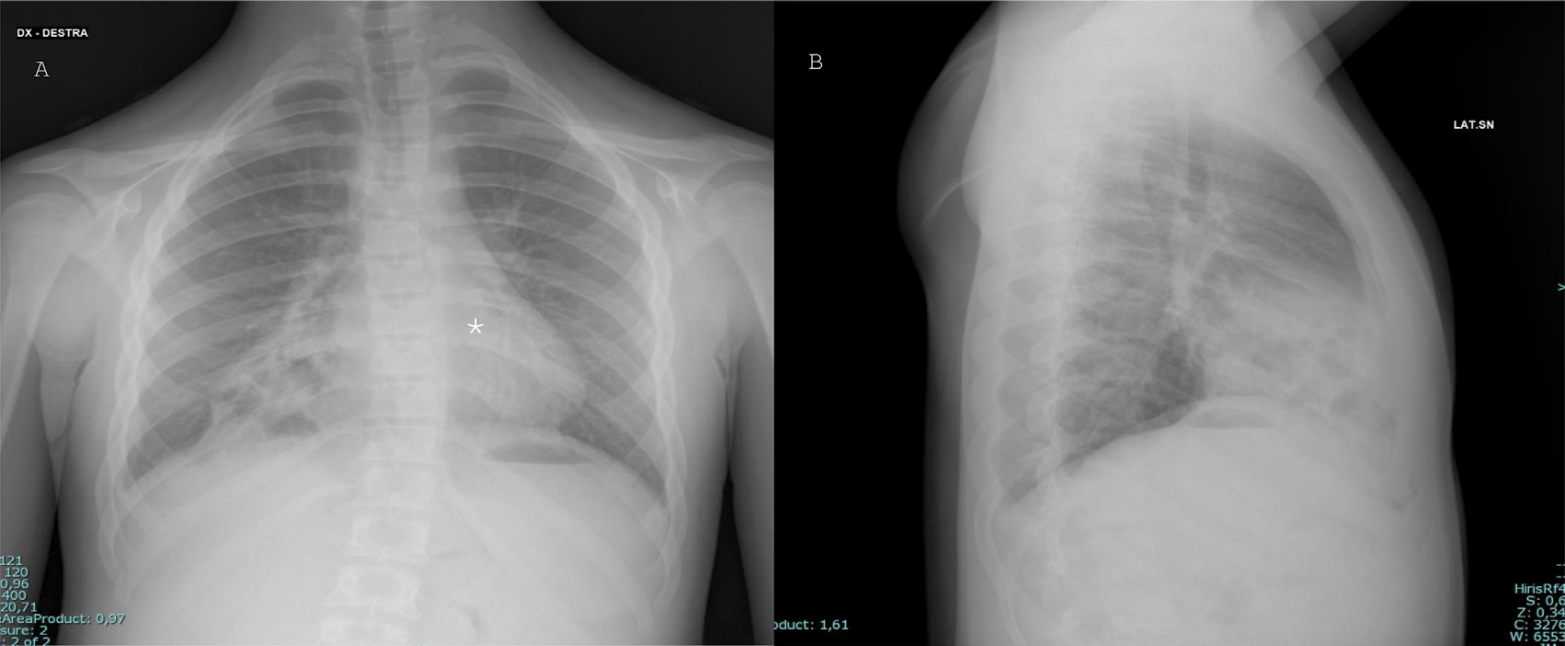
PA (A) and LL (B) projections chest x-ray showing showed multiple round areas of “bubble-like” increased transparency amidst a nonhomogeneous and blurred reduced transparency in the right basal paracardiac area (asterisk), a radiologic appearance that could be called “Bubbly heart”.

**Fig. 2:**
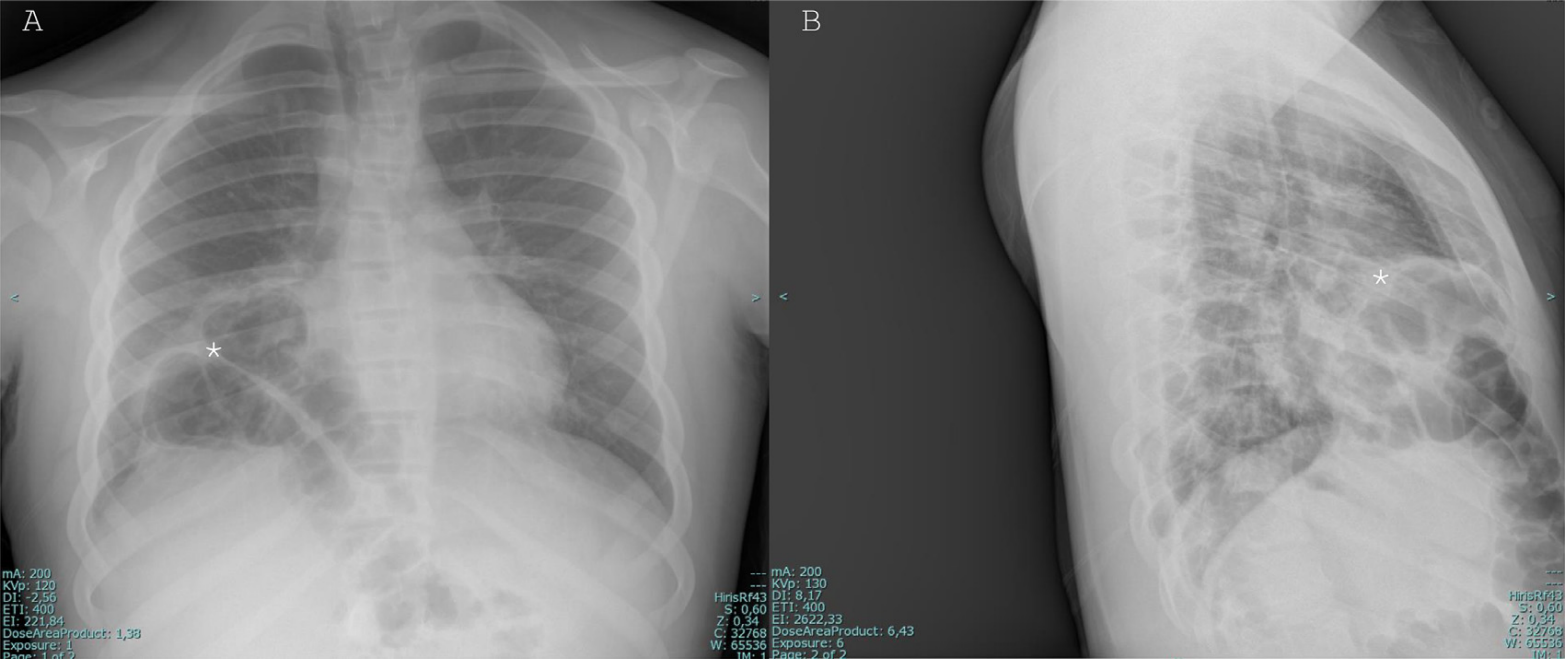
PA (A) and LL (B) projections chest x-ray showing bowel loops in the right pleural cavity. The evidence of haustras (asterisk) is consistent with herniation of trasverse colon.

**Fig. 3:**
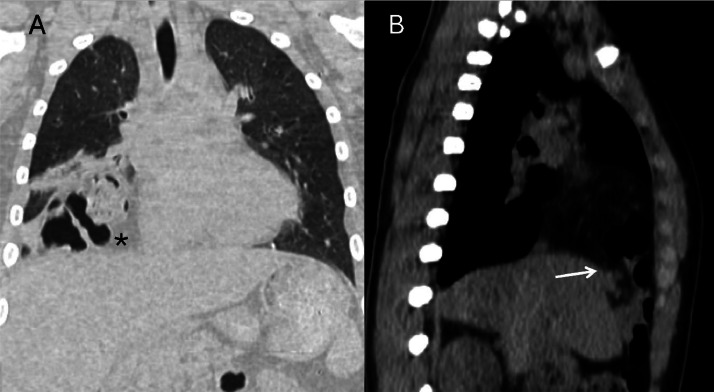
Computed tomographic scan (A: coronal plane; B: sagittal plane) showing bowel loops and omentum (asterisk) herniating through the right side of the diaphragm. In (B) the diaphragmatic defect is clearly seen, allowing to assess its extension (white arrow).

A 12-year-old young boy with Down syndrome came to the emergency room of our hospital for fever and shortness of breath. The patient underwent a chest x-ray that showed multiple round areas of “bubble-like” increased transparency amidst a nonhomogeneous and blurred reduced transparency in the right basal paracardiac area, a radiologic appearance that could be defined “Bubbly heart” (Fig. 1). Based on imaging and clinical characteristics, inflammation or abscess was suspected. The patient was discharged from the hospital and continued the prescribed antibiotic therapy at home. After 2 years the patient returned to our emergency room because of fever and dyspnea, the latter of which randomly and occasionally occurred in this period, more or less severe: another chest-x-ray was performed and showed an elevation of right-anterior hemidiaphragm’s profile with thoracic bowel displacement (pertaining of transverse colon was suspected for the evidence of haustras and for clear continuity with a median subdiaphragmatic colonic loop) with associated reduced transparency of the contiguous lung parenchyma, which given the fever could have been a sign of an infectious state (Fig. 2). Diaphragmatic hernia was suspected, and the diagnosis was confirmed on the following chest computed tomography (CT) scan, which clearly depicted the herniated transverse colon, along with mesenteric adipose tissue (Fig. 3).

Furthermore, CT cleared that the suspected parenchymal paracardiac inflammatory alteration seen in radiography was atelectasis and assured on the absence of hernia's complications, serving at the same time for preoperative treatment plan by thoracic surgeons depicting a diaphragmatic defect of 51 mm and its precise location.

## Discussion

MH is a rare type of congenital diaphragmatic hernia characterized by herniation through the foramen of Morgagni of omental fat, colon (most frequently transverse colon, as in our case) and rarely stomach, small intestine and liver [[3], [4], [5], [6], [7], [8]]. This kind of hernia can be associated with other congenital anomalies such as cardiac defects, trisomy 21 (like in our patient), intestinal malrotations, and pentalogy of Cantrell [[4], [5], [6], [7], [8], [9], [10], [11], [12], [13]]. It is usually asymptomatic, while pediatric patients can present with respiratory manifestations, such as recurrent lung infections, and gastrointestinal symptoms, all nonspecific symptoms that can delay diagnosis [[5], [6], [7], [8], [9],^10^]. Most of the time, detection and suspicion of this condition is made radiologically, including an anterior-posterior chest radiograph and a lateral one, and often the diagnosis is confirmed with a chest CT scan [14].

When, as in the first radiography of our case, both clinical symptoms and diagnostic images are nonspecific, the risk of missing the diagnosis and delaying the proper treatment, with the possible occurrence of future complications, is high.

Therefore, in a proper clinical setting, as the presence of reduced capability of the patient in describing his symptoms for speaking impairment (both mental and physical, i.e. tracheostomy or syndromic or autistic patients), the presence of a sign like the “bubbly heart,” although nonspecific, can be the only clue to get to the final diagnosis, thus sparing 2 years of both respiratory and gastrointestinal discomfort like in our case.

These diagnostic techniques give information about defect location and hernia involvement. CT with iodinated contrast agent injection is the gold standard for depicting any complications such as volvulus, incarceration, obstruction, and necrosis [[6], [7], [8], [9], [10], [11],[12], [13], [14]]. Treatment is surgical, even in asymptomatic patients, in order to prevent complications [15].

Because of the rarity of this condition, there are no guidelines indicating the best surgical treatment [9]. Surgical approaches may be open abdominal via laparotomy (favored in complicated cases as intra-abdominal adhesions, in emergent cases with respiratory insufficiency) [16], open thoracic via median sternotomy or thoracotomy (in large right-sided MH) [[17], [18]]; minimally invasive techniques, as laparoscopy or thoracoscopy, are the best approach in uncomplicated cases [[8], [9], [10], [11], [12], [13], [14], [15], [16], [17], [18], [19],^20^].

Prognosis is good, with a low risk of recurrence, although the recurrence rate is higher in patients with Down syndrome [4].

## Conclusion

MH is an uncommon defect, accounting only for 2% to 5% of congenital diaphragmatic hernias. Accompanying abnormalities, like Down syndrome, may be present. In this case, diagnosis may be delayed both due to the nonspecific clinical presentation and the syndromic patient’s condition. Even a nonspecific sign like the “Bubbly heart” appearance, in the proper clinical setting, can aid radiologists as a red flag to include MH in the differential diagnosis of patients with Down syndrome presenting with respiratory distress.

## Patient consent

Informed consent was obtained from the patient’s parents for the publication of this case.
